# The efficacy of public health information for encouraging radon gas awareness and testing varies by audience age, sex and profession

**DOI:** 10.1038/s41598-021-91479-7

**Published:** 2021-06-07

**Authors:** Natasha L. Cholowsky, Jesse L. Irvine, Justin A. Simms, Dustin D. Pearson, Weston R. Jacques, Cheryl. E. Peters, Aaron A. Goodarzi, Linda E. Carlson

**Affiliations:** 1 Departments of Biochemistry and Molecular Biology and Oncology, Robson DNA Science Centre, Charbonneau Cancer Institute, Cumming School of Medicine, University of Calgary, Calgary, AB Canada; 2 Faculty of Medicine, University of Saskatchewan, Saskatoon, SK Canada; 3 Cancer Epidemiology & Prevention Research, Alberta Health Services and Departments of Oncology & Community Health Sciences, Cumming School of Medicine, University of Calgary, Calgary, AB Canada; 4 Division of Psychosocial Oncology, Department of Oncology, Charbonneau Cancer Institute, Cumming School of Medicine, University of Calgary, Calgary, AB Canada

**Keywords:** Public health, Psychology and behaviour

## Abstract

Radioactive radon inhalation is a leading cause of lung cancer and underlies an ongoing public health crisis. Radon exposure prevention strategies typically begin by informing populations about health effects, and their initial efficacy is measured by how well and how fast information convinces individuals to test properties. This communication process is rarely individualized, and there is little understanding if messages impact diverse demographics equally. Here, we explored how 2,390 people interested in radon testing differed in their reaction to radon's public health information and their subsequent decision to test. Only 20% were prompted to radon test after 1 encounter with awareness information, while 65% required 2–5 encounters over several months, and 15% needed 6 to > 10 encounters over many years. People who most delayed testing were more likely to be men or involved in engineering, architecture, real estate and/or physical science-related professions. Social pressures were not a major factor influencing radon testing. People who were the least worried about radon health risks were older and/or men, while negative emotional responses to awareness information were reported more by younger people, women and/or parents. This highlights the importance of developing targeted demographic messaging to create effective radon exposure prevention strategies.

## Introduction

Lung cancer in people who have never smoked is currently the 7th leading cause of cancer-linked mortality globally, and the incidence continues to increase^1–6^. The repetitive inhalation of radioactive radon-222 (^222^Rn) gas is the foremost cause of lung cancer in North America and Europe among people who have never smoked and causes many thousands of new diagnoses and related deaths per year^7,10–16^. Radon is also a major factor underlying lung cancer in smokers due to the synergistic effects of these two exposures^4,7–16^. The *International Agency for Research on Cancer* classifies radon as a category 1 carcinogen, as it discharges alpha particle ionizing radiation that damages lung cell DNA to produce genetic mutations that drive cancer formation^15–17^. The radiation from radon is measured in Becquerels (Bq) per cubic meter (m^3^), corresponding to one alpha particle emission per second per cubic metre of air. Relative lifetime risk of lung cancer rises by 16% per ≥ 100 Bq/m^3^ long term radon exposure^18,19^. While radon is generated deep underground across most of the world’s geology, it is important to acknowledge that hazardous radon exposure is a relatively recent, human-made problem, as it otherwise dilutes naturally and quickly in the atmosphere to low levels with no observable health impacts^15^. Unfortunately, twentieth to twenty-first century construction practices have produced many buildings that capture, contain and concentrate radon to unsafe levels. In North America particularly, this continues to worsen as newer residential buildings are constructed increasing innate risks of producing high radon levels that are disproportionately impacting younger individuals with children^12,13,20^. Western Canada, for example, has been identified as a region of excessive residential radon gas exposure, with long term inhalation being responsible for approximately 1 new lung cancer case per day in some provinces^2,7–9,11^.

Historically, radon prevention strategies have focussed on convincing individuals to test buildings and personally invest in mitigation solutions if the building exceeds certain administrative radon levels (e.g. 200 Bq/m^3^ in Canada or many European countries, 148 Bq/m^3^ in the USA)^21,22^. The responsibility for these efforts is broadly split between government or health authority-sponsored testing programs, non-profit and/or citizen science-based testing advocates, and for-profit private industry. These groups often employ similar communication methods that typically begin by widely broadcasting radon exposure health effects through messaging targeting the general public (examples in Supplemental Information). The overall efficacy of a given strategy is measured by how well and how fast the message is able to persuade individuals to take action, i.e., obtain a radon test and successfully carry it out. Following testing, related and often additional follow-up messaging encourages individuals to mitigate properties if necessary. In this message-to-action paradigm, there is little understanding if these ‘one-size-fits all’ strategies of communicating radon health risks are equally effective across distinct psychosocial demographics. In this respect, information on the efficacy of previous radon program is still emerging and limited, with some studies reporting broad radon awareness outcomes as a function of ‘classic’ radon programs in regions spanning Europe and North America^23–30^. However, in the absence of detailed data on this topic, it is challenging to ‘individualize’ and develop radon exposure prevention programs in an evidence-informed manner.

Evaluating how diverse groups of people respond to radon awareness information is vital to improving exposure reduction strategies for several reasons. Firstly, to better promote inclusivity, public health investments should target the widest possible populations without excluding or under-serving disadvantaged groups. Secondly, to improve the “message-to-action efficacy” of public radon reduction programs, it is important that they be based on the best available evidence, as approaches over the past > 30 years have not achieved desired outcomes. In Canada, for example, despite decades of public investment in radon information and reduction programs, fewer than 6% of residential properties have been radon tested, and both radon levels in new properties and lung cancer incidence in never-smokers are at historic highs^12,13,20,21^. This is especially poignant when set against lung cancer prevention programs for tobacco exposure: a potent, preventable carcinogen similar to radon, but for which large-scale population exposure prevention through public health campaigns have been remarkably successful^31^, and has included investment in behavior-change research. To start to address key knowledge gaps, our objective was to explore associations between age, sex, profession, and other demographics, and how people encounter and respond emotionally to radon awareness information. Furthermore, we assessed how these demographics influence personal perceptions of radon knowledge and, most importantly, motivation for taking action (i.e., radon testing).

## Results

### Study recruitment, cohort details and demographic distribution

Our total study group encompassed 18,971 Canadians who, following informed consent into a citizen science-based non-profit radon testing program, opted to perform long-term alpha track radon tests within their residential properties between 2015–2020. The full details of this cohort and their radon test outcomes were detailed recently^20^. Of the main cohort, 7,481 Western Canadian participants who were active within the study in 2018 were invited to retrospectively (in relation to completing a radon test) provide demographic and psychosocial survey responses to questions concerning their radon awareness experience and decision to test. Of these, 2,390 individuals provided complete responses (32% participation). For this analysis, we considered: age, ‘biological sex at birth’, parental status, relationship status, employment status, status in professions deemed to potentially have specialist knowledge of radon (including public health, industrial hygiene, architecture, real estate, engineering, physical sciences (geology, chemistry, physics), or medicine and health or life sciences), and experience with cancer. Response distributions for the entire cohort are summarized in Supplemental Figure 1.

It is important to note that the data we collected was in the context of ‘sex at birth’ and was largely binary in nature, although participants were provided with the option to select “other” or “prefer not to say” (i.e., a sex unspecified by the participant). Please note that we will use the terms 'men', 'women' and 'unspecified' for ease of reading, and to describe this in the context of this study. In brief, the cohort comprised of 42.9% women, 56.1% men and 1% unspecified, and was largely balanced except for a modest skew in those over the age of 60 towards being men (Figure S1A). The mean age of women was 49.2 y, while men were 53.0 y. 73% of participants had at least one child (Figure S1B), 68% were in full or part-time employment (Figure S1C), and 88% were in a long-term relationship (Figure S1D). A majority (53.8%) had no current or previous association with a profession deemed to have specialist radon knowledge (Figure S1E). Only 15.8% reported a personal experience with cancer (i.e., they or another immediate member of the household had/has cancer), while 63.4% of participants reported a known family history of any cancer (i.e., a known incidence of cancer within the participants’ living or non-living family, excluding themselves and immediate members of the household) (Figure S1F). Complete questionnaires and response options are provided in Supplemental Information, Section I.

### Age, sex and profession influences how people first encounter radon awareness information

**Figure 1:**
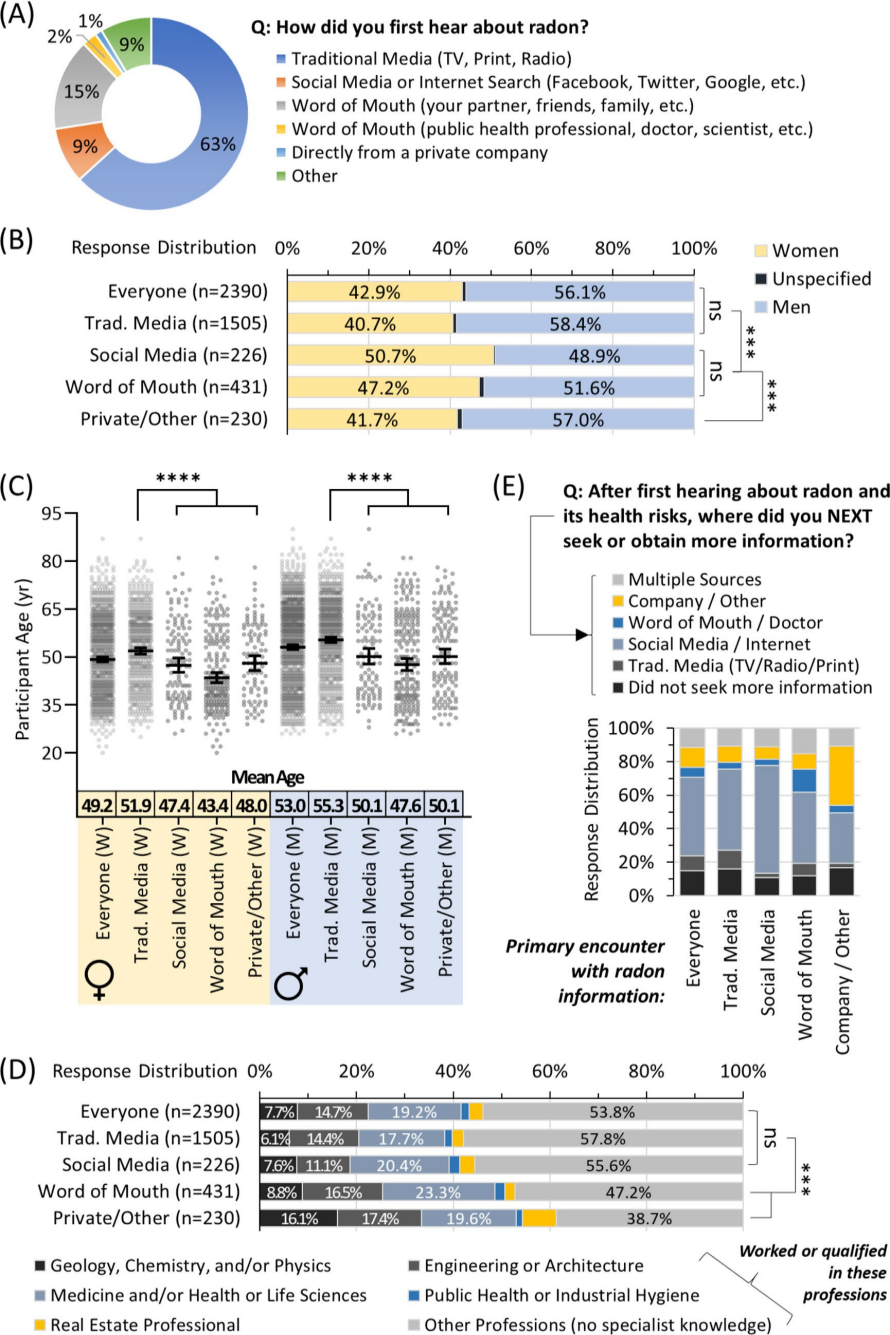
Age, sex, and profession influence how people first encounter radon awareness information. Panel (**A**) Overall distribution of responses for the first encounter with radon awareness information. Panel (**B**) First encounter with radon awareness information as a function of sex. Panel (**C**) First encounter with radon awareness information as a function of sex and age. Mean Age refers to a geometric mean ± CI95%. Panel (**D**) First encounter with radon awareness information as a function of status (worked or qualified) in professions with or without the potential for a specialist on radon. Panel (**E**) Response distribution of how groups based on their first encounter with radon awareness information went on to next seek or obtain more information. Statistical comparisons are Mann–Whitney pairwise nonparametric t-tests of comparisons for scatter plot data or 1-way ANOVA for all other data. **** = p < 0.0001; *** = p < 0.001; ns = p > 0.05. Figures were prepared using Excel and GraphPad Prism 9.1.1 (225) (www.graphpad.com).

We categorized how an individual’s first encounter with radon awareness information occurred (Fig. 1A). Traditional media (tv, print, radio) was the most prevalent, followed by word of mouth, social media/the internet, an unspecified ‘other’ and, lastly, information from private companies. Women were moderately but significantly (p < 0.001) more likely to use word-of-mouth and social media as a point of the first interaction with radon information (Fig. 1B). For both sexes, being younger significantly (p < 0.0001) increased the likelihood of using social media / the internet, or word of mouth as the mode of the first encounter with radon information, while those relying on traditional media were older (Fig. 1C). For those in professions with potential specialist radon knowledge, those in engineering, architecture, real estate, and/or the physical sciences were more likely to have first encountered radon information via private company or unspecified means, whilst those in medicine, health or life sciences were most likely to have used word-of-mouth (Fig. 1D). Traditional media was the most common point of the first contact for the majority without specialist knowledge of radon. There were no significant (p > 0.05) differences in the mode of the first encounter with radon information based on an individual’s cancer experience, or parental, relationship or employment status. We next categorized how people obtained further information as a function of their first encounter. Except for the 10% who first encountered radon information from a private company or unspecified means, the remaining majority primarily used social media/internet to expand their knowledge, irrespective of the mode of first encounter (Figs. 1A,E).

### Radon knowledge perceptions differ by profession and sex but not age

**Figure 2:**
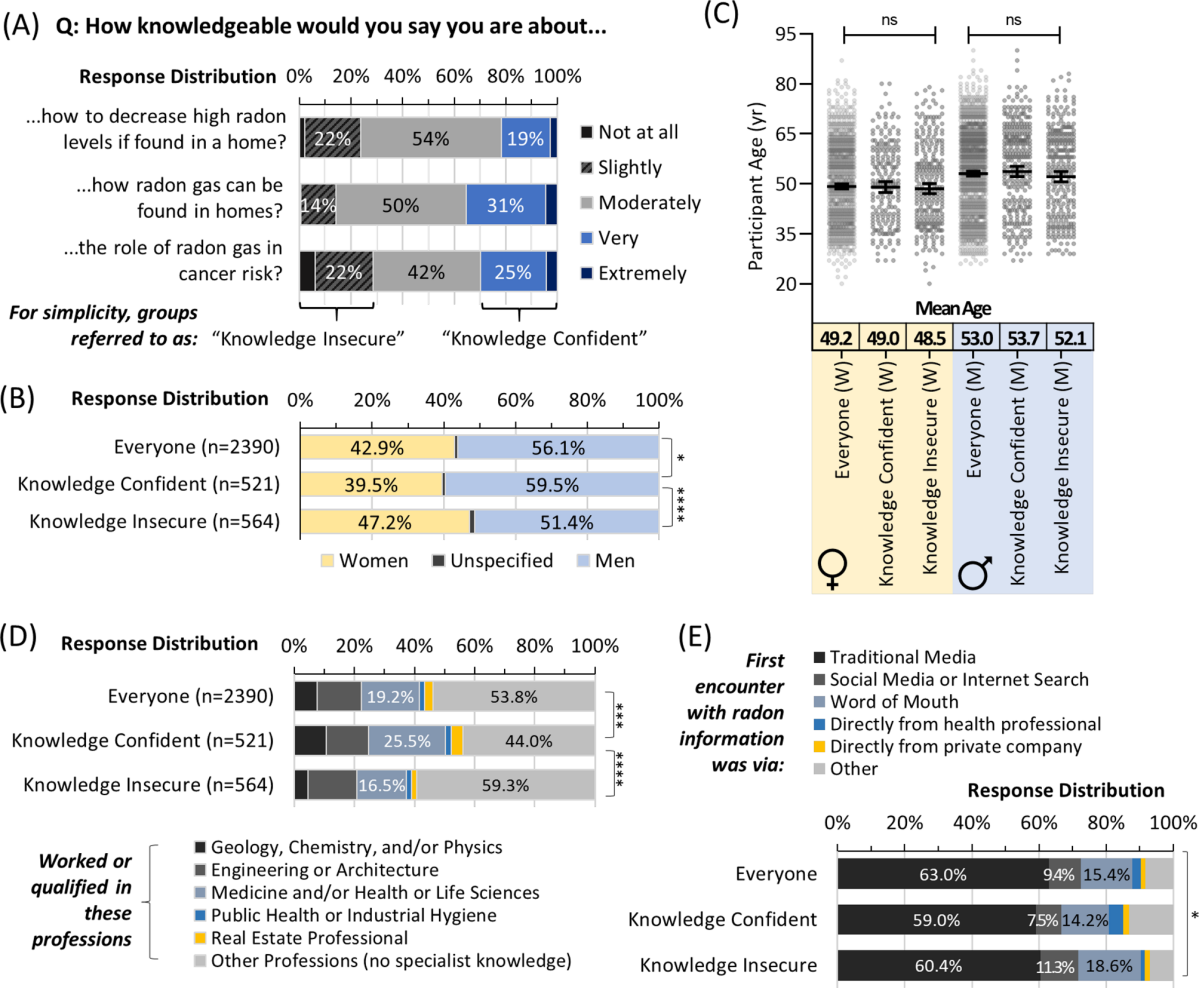
Radon knowledge perceptions differ by profession and sex but not age. Panel (**A**) Overall distribution of responses to indicated questions on self-perceptions of radon knowledge. Panel (**B**) Self-perceptions of radon knowledge as a function of sex. Panel (**C**) Self-perceptions of radon knowledge as a function of sex and age. Mean Age refers to a geometric mean ± CI_95%_. Panel (**D**) Self-perceptions of radon knowledge as a function of status (worked or qualified) in professions with or without the potential for a specialist on radon. Panel (**E**) Self-perceptions of radon knowledge as a function of the first encounter with radon awareness information. Statistical comparisons are Mann–Whitney pairwise nonparametric t-tests of comparisons for scatter plot data or 1-way ANOVA for all other data. **** = p < 0.0001; *** = p < 0.001; * = p < 0.05; ns = p > 0.05. Figures were prepared using Excel and GraphPad Prism 9.1.1 (225) (www.graphpad.com).

We next evaluated self-perceptions of knowledge regarding: (i) the role of radon in cancer risk, (ii) how it can be found in homes, and (iii) how to decrease high levels if present, using a standard 5-point Likert scale (Fig. 2A). About half rated themselves as having moderate knowledge, with the remainder splitting between very to extremely high knowledge (for simplicity = ‘knowledge confident’) or slight to no knowledge (for simplicity = ‘knowledge insecure’). Men were more likely to express confidence, while women were slightly but significantly (p < 0.0001) more likely to express knowledge insecurity (Fig. 2B). Age was not a significant (p > 0.05) factor (Fig. 2C), while, as expected, experience or qualifications in a profession with potential specialist radon knowledge showed a positive relationship with knowledge and was significant (p < 0.0001) (Fig. 2D). Radon knowledge perceptions were largely the same as a function of the mode of the first encounter with radon information, with a small but significant (p < 0.05) increase in confidence for those obtaining information from a health professional or unspecified means (Fig. 2E). There were no significant (p > 0.05) differences in knowledge perceptions based on an individual’s cancer experience, or parental, relationship or employment status.

### Emotional reactions to radon awareness vary by personal demographics, but not a first encounter

**Figure 3:**
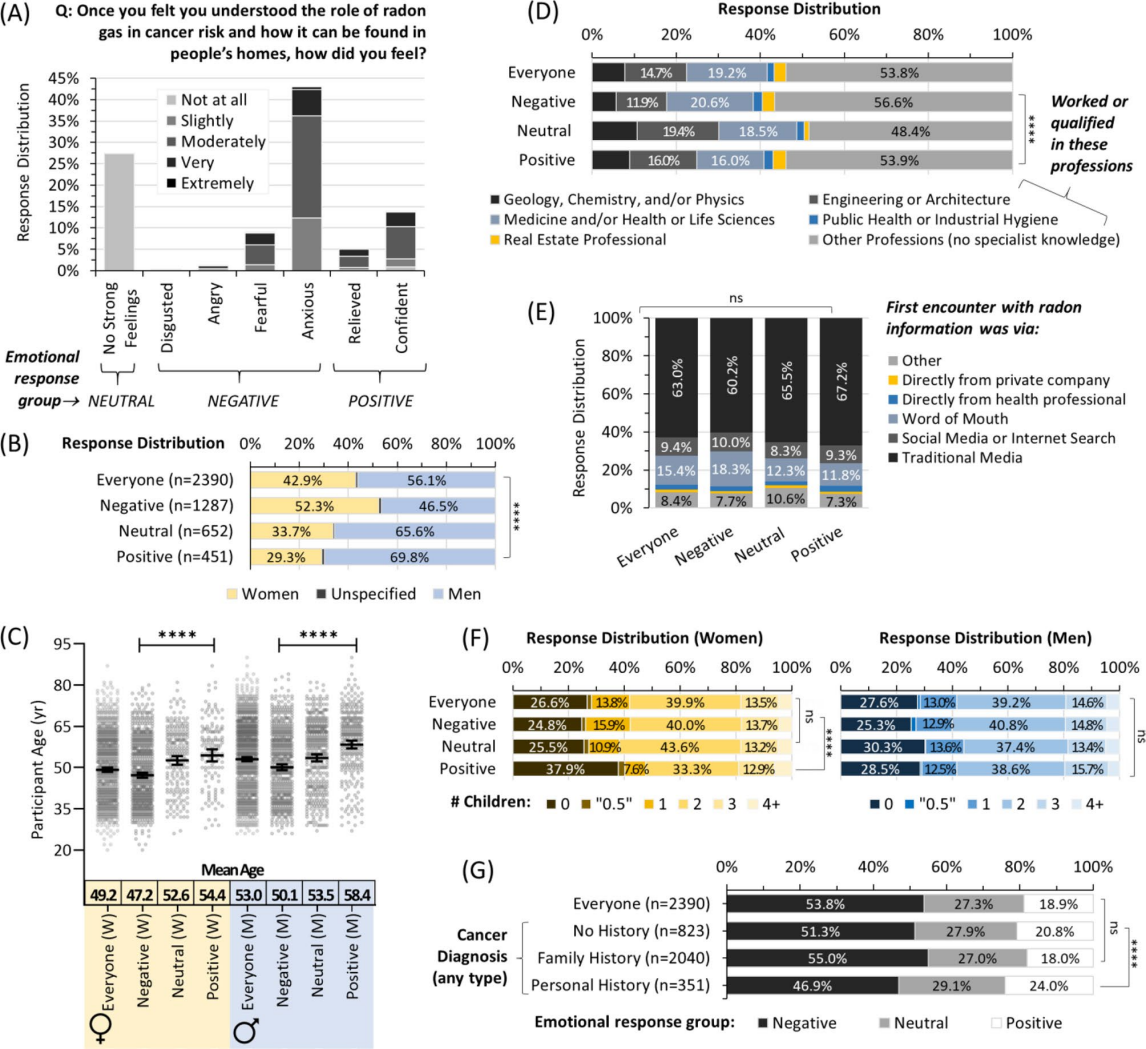
Emotional reactions to radon awareness vary by personal demographics, but not the first encounter. Panel (**A**) Overall distribution and Likert scale intensity of emotional responses to gaining awareness of radon health effects and how it can be found in homes. Panel (**B**) Emotional response distributions from (**A**) as a function of sex. Panel (**C**) Emotional response distributions from (**A**) as a function of sex and age. Mean Age refers to a geometric mean ± CI95%. Panel (**D**) Emotional response distributions from (**A**) as a function of status (worked or qualified) in professions with or without the potential for a specialist on radon. Panel (**E**) Emotional response distributions from (**A**) as a function of their first encounter with radon awareness information. Panel (**F**) Emotional response distributions from (**A**) for parental status of women (left, yellow) and men (right, blue). Panel (**G**) Emotional response distributions from (**A**) as a function of experience with a cancer (of any type) diagnosis. Statistical comparisons are Mann–Whitney pairwise nonparametric t-tests of comparisons for scatter plot data or 1-way ANOVA for all other data. **** = p < 0.0001; ns = p > 0.05. Figures were prepared using Excel and GraphPad Prism 9.1.1 (225) (www.graphpad.com).

To assess emotional reactions to gaining radon awareness, we next asked participants to reflect on their feelings once they felt they understood the role of radon gas in homes and how it relates to cancer risk (Fig. 3A). Once again, a standard 5-point psychometric Likert scale was applied. 72.6% of participants reported strong feelings, of which 53.8% were ‘negative’ (disgust, anger, fear, or anxiety), and 18.8% were ‘positive’ (confidence, relief). Women were significantly (p < 0.0001) more likely to report negative feelings (Fig. 3B). For both sexes, older individuals were more likely to have neutral or positive feelings after gaining radon awareness, while those experiencing negative emotional responses were significantly (p < 0.0001) younger (Fig. 3C). There were also significant (p < 0.0001) differences in emotional responses as a function of professional experience, with those in engineering, architecture, real estate, and/or the physical sciences more likely to report no strong feelings, relief, or confidence (Fig. 3D). There were no significant (p > 0.05) differences in emotional responses based on the distribution of point of the first encounter with radon awareness information (Fig. 3E), or an individual’s relationship or employment status (data not shown). For women only, positive emotional outcomes were more likely in those with no children (Fig. 3F). Somewhat surprisingly, personal history with cancer correlated to a modest increase in neutral or positive emotional outcomes relative to other groups (Fig. 3G).

### Time to action following radon awareness diverges strongly by sex and profession

**Figure 4:**
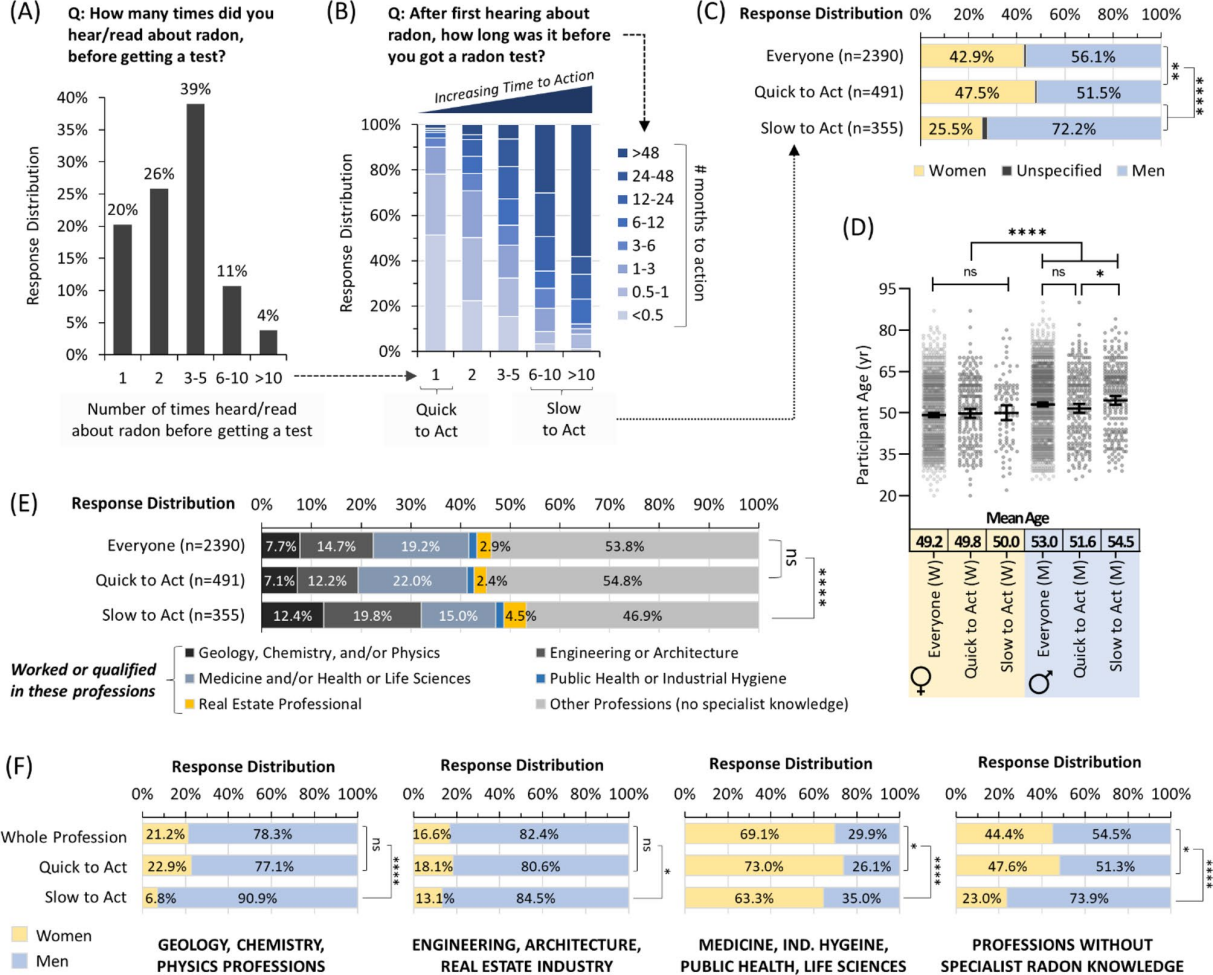
Time to action following radon awareness diverges strongly by sex and profession. Panel (**A**) Overall distribution of responses for the number of times people report encountering radon awareness information before obtaining a radon test. Panel (**B**) The amount of time that people report between first encountering radon awareness information and obtaining a radon test is expressed as a function of data in (**A**). Panel (**C**) Time to action (from **A**,**B**) as a function of sex. Panel (**D**) Time to action (from **A**,**B**) as a function of sex and age. Mean Age refers to a geometric mean ± CI95%. Panel (**E**) Time to action (from **A**,**B**) as a function of status (worked or qualified) in professions with or without the potential for specialist knowledge on radon. Panel (**F**) Time to action (from **A**,**B**) was expressed as in (**C**) but split into the professional alignment groups from (**E**). Statistical comparisons are Mann–Whitney pairwise nonparametric t-tests of comparisons for scatter plot data or 1-way ANOVA for all other data. **** = p < 0.0001; ** = p < 0.01; * = p < 0.05; ns = p > 0.05. Figures were prepared using Excel and GraphPad Prism 9.1.1 (225) (www.graphpad.com).

Our next goal was to assess the cohort’s relative speed or delay in obtaining a radon test kit (i.e., ‘the time to action’) after becoming radon aware (i.e., understanding ‘the message’). We measured this by recording how many times an individual heard or read about radon before getting a test, and the number of months over which this took place. Only 20% of individuals obtained a radon test kit after a single encounter with radon awareness information (Fig. 4A). 65% required between 2 and 5 interactions, while 11% required 6–10 encounters and another 4% needed > 10 distinct points of radon information contact before getting a test kit. The number of months to action corresponded well with delay to action, with 90% of the most amenable (1 interaction before getting a test) group requiring less than 3 months to get a kit, and 90% of those who delayed action the most (≥ 6 interactions before getting a test) taking anywhere from 6 months to > 4 years (Fig. 4B). Men were substantially and significantly (p < 0.0001) more likely to delay action, while women were somewhat quicker to act (Fig. 4C). Age was not a significant factor in the delay of action for women, although younger men took action slightly faster compared to older men (p < 0.05) (Fig. 4D). Professional alignment to real estate, the physical sciences, engineering, or architecture corresponded to a substantial increase in time to action (Fig. 4E). To account for sex ratio imbalances within each professional grouping, we combined datasets in Fig. 4C,E. We observed that the greater time to action of engineering, architecture, or real estate was largely independent of sex (Fig. 4F). By contrast, greater time to action in medicine, industrial hygiene and the physical or life sciences was more likely in men. For most participants without specialist radon knowledge, men were significantly (p < 0.0001) and substantially more likely to wait longer to obtain a radon test. There were no significant (p > 0.05) differences in the delay to action based on an individual’s cancer experience, or parental, relationship or employment status.

### For most people, social pressures and landlords do not motivate radon testing

**Figure 5:**
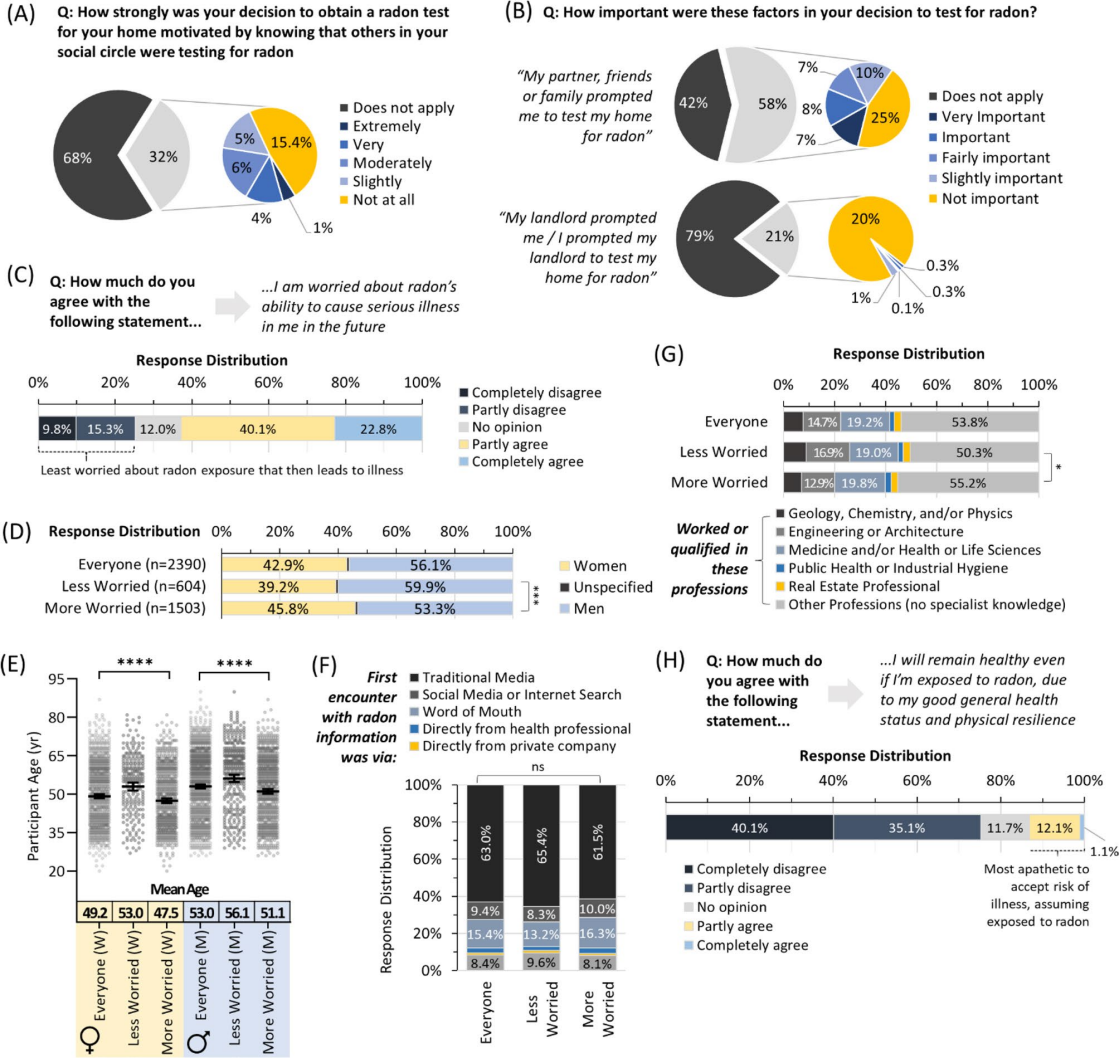
Social pressures do not motivate radon testing, and risk perceptions vary primarily by age. Panel (**A**) Overall distribution of responses assessing role of general social circle pressure in the decision to radon test. Panel (**B**) Response distributions assess immediate family, friend and landlord-related social pressure in decision to radon test. Panel (**C**) Overall distribution of Likert scale responses about concern of future radon exposure leading to illness. Panel (**D**) Relative concern about radon exposure leading to illness (as in **C**) as a function of sex. Panel (**E**) Relative concern about radon exposure leading to illness (as in **C**) as a function of sex and age. Mean Age refers to a geometric mean ± CI95%. Panel (**F**) Relative concern about radon exposure leading to illness (as in **C**) as a function of their first encounter with radon awareness information. Panel (**G**) Relative concern about radon exposure leading to illness (as in **C**) as a function of status (worked or qualified) in professions with or without the potential for a specialist on radon. Panel (**H**) Overall distribution of Likert scale responses to self-perceptions of future risk of illness assuming exposure to radon. Statistical comparisons are Mann–Whitney pairwise nonparametric t-tests of comparisons for scatter plot data, or 1-way ANOVA for all other data. ****p < 0.0001; ***p < 0.001; * = p < 0.05; ns = p > 0.05. Figures were prepared using Excel and GraphPad Prism 9.1.1 (225) (www.graphpad.com).

To investigate radon testing and awareness motivations, we asked participants to report how strongly their decision to obtain a test was influenced by peer and social pressure factors, including: (i) knowing that those within their social circles were testing for radon, and (ii) how important prompting from friends, family, partners or landlords was to test. Once again, this was assessed using a 6-point Likert scale. Although a third of the cohort were aware that others in their social circles were radon testing, of these, only a small minority (5% of all participants) found this to be very or extremely important (Fig. 5A). Similarly, only 15% of all participants reported peer and social pressures from friends, family, or partners to be important or very important to their decision to obtain a radon test, while only 0.6% indicated their landlord factored into radon test decision making (Fig. 5B). There were no significant (p < 0.05) differences in any demographic variables between the minorities reporting the relevance of herd mentality, social pressures and/or landlords to their decision making, compared to the remaining majority.

### Worry about radon exposure, and radon-associated illness varies primarily by age

To assess relative concern about how future radon exposure might impact health, we asked participants to declare whether they were ‘*worried about radon’s ability to cause serious illness*’ in the future, on a 6-point Likert scale ranging from “not at all worried” to “extremely worried”. Outcomes were mixed, and while a total of 62.9% (40.1% partly, and 22.8% completely agreed they) were worried about radon risks as being pertinent to their future health, a total of 25.1% completely or partly disagreed with the statement and were less concerned about radon exposure as a pertinent future health risk (Fig. 5C). The group who worried less about radon health risks was somewhat over-represented by men (p < 0.001) and, for both sexes, was significantly (p < 0.0001) older (Fig. 5D,E). There were no significant (p > 0.05) differences in relative concern based on their point of first encounter with radon awareness information (Fig. 5F) or their relationship, parental or employment status. A significant (p < 0.05) but very small decrease in worry was noted for those aligning to a profession likely to have specialist radon knowledge (Fig. 5G).

To separate perceptions of future radon exposure from concerns of radon-induced illness if exposure were assumed to have taken place, we asked participants to declare whether they felt they would ‘*remain healthy even if…exposed to radon, due to [their] general good health status and physical resilience*’, on a 6-point Likert scale with endpoints of “strongly disagree” to “strongly agree”. In this scenario, a more substantial majority (75.2%) of participants fully or completely agreed that radon exposure (if verified to have occurred) would impact their health, with only 12.1% partly disagreeing and 1.1% entirely disagreeing (Fig. 5H). Curiously, none of the demographics for this minority (13.2%, those who were hesitant to perceive verified radon exposure as being pertinent to their future health) differed significantly (p > 0.05) from the overall cohort or those of opposing views.

## Discussion

Understanding how different groups of people encounter, react to, and variably take action after gaining radon awareness is foundational knowledge needed to improve public health programs aimed at reducing radon gas exposure in large populations. As a first major step towards this, we have considered several primary demographics—most notably age, sex, and profession—in relation to the radon testing paradigm and find that all three are highly relevant variables that influence the speed and ease with which individuals opt to radon test a residential property after encountering public health information. By considering each variable in the context of psychological knowledge of risk communication and cognitive processing of information, several key recommendations can be made for the attention of public health, academic and private groups engaged in radon awareness activities.

Younger people were more likely to experience negative emotional reactions and be more worried about long-term health consequences of radon, while older individuals downplayed risks. Strictly speaking, both groups' responses are appropriate, as increased age at time of radon exposure (especially ≥ 65 years old) is indeed correlative with reduced relative risk of lung cancer^32^.

This perhaps also explains why people with a personal history of cancer (i.e., they or a member of household has or had cancer) are more likely to react positively to gaining radon awareness, as increased age is the greatest predictor of cancer risk^33,34^. We also find that younger people encounter radon awareness more often via social media and/or word-of-mouth means of communication. While predictable^35^, this finding becomes pivotal when one considers that younger people are more susceptible to negative health consequences from exposure. At least in North America, they are more likely to experience greater overall radon doses due to biases within the built environment^11–13,20,21,32,36,37^. As targeting younger people (especially those with children), is crucial for achieving the greatest reduction in population-based relative risk of lung cancer, one key recommendation from our work is for radon awareness strategies to increase investment in social media-based and/or word-of-mouth oriented communication techniques that better reach younger demographics (e.g., influencer campaigns).

In the context of how people find and react to public health information, sex matters^38^. We find women are more likely to utilize social media and other interactive means as a point of the first contact with health information on radon gas, were more likely be concerned (worry) about radon, and experienced more negative emotional reactions in response to gaining awareness. It is probable that these underlie the increased willingness of women to obtain a residential radon test, in terms of both greater speed and fewer information encounters required to prompt action. This notion is supported by previous work documenting that, in 1990, American women were more likely (than men) to express the intent to perform a radon test^39^. By contrast, men were far more challenging, taking substantially longer to obtain a test, and feeling less anxiety and worry about the potential risks of radon exposure. From a health outcome perspective, these differences mean that people in households where men are the primary radon test decision-maker are more likely to experience longer radon exposure durations, and thus a greater overall particle radiation dose to the lungs, potentially increasing relative risk of occupants to developing lung cancer. Hence, to improve population-based outcomes and the return on public health investment, our work suggests that radon awareness material could be directed preferentially at women to improve the speed and ease of encouraging radon testing. As with strategies that better target younger people, this means once again that leveraging social medial and word-of-mouth methods are likely to have the greatest future impact. Equally, investing additional resources in new message content and/or modes of communication that better convince men to radon test may have similar outcomes.

One somewhat surprising outcome of our work was the influence that professional background had on the efficacy of radon awareness information in promoting testing. For those who are qualified or have worked in medicine, life sciences, public health and/or industrial hygiene, their relative amenability to radon testing is logical, in that this is a group with probable first-hand knowledge of oncology, cancer origins and/or consequences, may have participated in radon-related public health campaigning, and/or are very over-represented by women^40^. However, it is less apparent why those in the physical sciences, engineering, architecture and/or real estate would require more encounters with radon awareness information and be much slower to obtain a test. In these cases, specialized knowledge of radon would be less likely to be linked to health, but rather locations and physical properties of radon-generating radionuclides for the physical sciences and logistical concerns or administrative annoyances for engineers, architects, and real estate brokers. Although certainly these professions are over-represented by men^41^, this alone does not explain the bias as women from these groups—particularly engineering, architecture and/or real estate—were similarly slow to act. Rather, we suggest that this is potentially a manifestation of cognitive dissonance.

Cognitive dissonance theory postulates that individuals are uncomfortable holding opposing beliefs about themselves. This causes feelings of discomfort and increases motivation to relive the dissonance, usually by changing one belief or the other. The opposing beliefs may be “*I am a good person who wants to help others*”, and “*I work in a profession that I believe might partially cause or exacerbate lung cancer *via* radon exposure*” (which we stress is not true) or even, more simply, “*this large problem is not something I am prepared to handle*”. To relive the dissonance, and in the immediate absence of convincing contrary information, it is easier and less threatening to self-esteem to change the latter vs. the former belief, as the former is a reflection of core identity and therefore more threatening to change. Hence, it relieves the discomfort of dissonant cognitions if one convinces oneself that there is not really an increased risk of lung cancer due to radon exposure. This downplays risks and delays action on testing when confronted with radon awareness information. This idea is supported by work exploring reasons for not pursuing radon testing reported by people in Ireland^42^, and has been discussed in^43^. This is important to consider from two perspectives: (i) radon exposure durations lengthen as more time is required from encountering radon awareness material to obtaining a test, increasing exposure risk for those aligned with certain professions in a manner distinct from their actual occupational environment; and (ii) because people from the physical sciences, engineering, architecture and/or real estate have the potential to be influential change-makers for radon awareness and testing, but are not doing so.

In the future, it will be important to assess whether the recommendations we suggest have the desired outcomes, including: (i) whether social media influencer campaigns are useful to encourage younger people and/or women to become radon aware and test their residential environments; (ii) whether preferentially targeting women with existing material or catering to men with new communication tools elicits improved radon testing uptake; and (iii) if providing better resources to those in specific professions to alleviate cognitive dissonance effects results in shortening the time they require from the first encounter to radon testing, and whether such intervention could potentially transform this population into key advocates. It will be important to address some of the limitations of our work, including considering the impacts of gender identity, socioeconomic status, and cultural, ethnic, and regional disparities in attitudes and beliefs (which we have not yet explored). Like most radon awareness campaigns, our citizen-science based program requires some financial and time investment from participants when performing a radon test (albeit in a non-profit manner), and so we acknowledge some economic barriers to participation. However, balancing this to some extent is that our methods are still applicable to the limited public health campaigns where radon tests are dispersed at no cost. Another limitation is that we only included people who already conducted a radon test in their homes, so the attitudes and beliefs of those who have chosen *not* to test for radon as yet cannot be generalized.

Finally, the most important follow-up to this work will be to consider how diverse groups of people understand and react to their specific radon test outcomes, and how demographic variables influence the decision to mitigate a residential property for radon based on that outcome. This, ultimately, is the only actual action that will reduce radon exposure and reduce the future burden of radon-induced lung cancer.

## Methods

### Statement of approvals

All activities were pre-approved by the Conjoint Health Research Ethics Board, Research Services, University of Calgary (IDs = REB17-2239, REB19-1522) or the Health Research Ethics Board of Alberta, Cancer Committee (HREBA.CC-17-0246), adhering to citizen science research best practice^44^, and in accordance with all regional guidelines and regulations.

### Participant eligibility and enrollment

Enrollment was based on convenience recruitment for all wanting to join, with all adult homeowners and renters in any residential building type being eligible. No data from any constituent part of this cohort were from known or pre-selected lung cancer cases. Commercial offices or hospitality service buildings were not considered. Records of informed consent were obtained in all cases. Participants were permitted to withdraw at any time.

### Radon awareness communication

Communication methods for study recruitment included dissemination of radon awareness information and provision of opportunities for radon testing through print media, public seminars, online (website and social media) and traditional mass media via organic (unpaid) TV, magazine, and radio exposure in an untargeted manner. Each of these points of communication was further boosted in an organic (unpaid) manner by health agencies, non-profit groups, and some private organizations. Radon awareness content included basic, scientifically-informed facts on radon, its health effects in the context of lung cancer and highlighted both historic facts, figures, and emerging regional statistics (examples are outlined in Supplemental Information, Section II).

### Surveying

From 2015–2020, Canadians purchased alpha track 90 + day radon detectors that they then deployed, returned for analysis, and later received their specific radon reading in a confidential manner. Radon outcomes for this cohort were reported recently in^20^. Non-profit study kits were $51.99. Following consent and placement of a radon test, all participants active within the study in October of 2018 were invited to complete demographic surveys and questionnaires on radon awareness and testing experiences using the Qualtrics survey platforms.

### Statistical analysis

Statistical analyses were carried out using Excel and GraphPad Prism 9.1.1 (225) (www.graphpad.com). One way ANOVAs were carried out to test differences between groups (e.g., year of construction, occupant age, mSv, etc.), with Bonferroni-Holm corrected post-hoc testing carried out to characterize group differences for pairwise comparisons if the ANOVA reached significance. Mann–Whitney pairwise nonparametric t-tests were used to assess the significance of scatter plot data.

## Supplementary Information

Supplementary Information.

## Data Availability

The de-identified raw data sets generated by the current study are available to academic researchers at public institutions following reasonable requests to Dr. Goodarzi, and will require a legally-binding data transfer agreement. Data may not be used for private, commercial, or for-profit purposes for any reason.
