# Supplementary material for: The efficacy of public health information for encouraging radon gas awareness and testing varies by audience age, sex and profession
Source: Sci Rep. 2021 Jun 7;11:11906. doi: 10.1038/s41598-021-91479-7 (PMC8185097; doi:10.1038/s41598-021-91479-7)
Supplement: Supplementary file 1 — Supplementary Information. [file 41598_2021_91479_MOESM1_ESM.pdf]

SUPPLEMENTAL FILE

for

The efficacy of public health information for encouraging radon gas awareness and testing varies by audience age, sex and profession

Natasha L. Cholowsky[1*], Jesse L. Irvine[1*], Justin A. Simms[2], Dustin D. Pearson[1], Weston R. Jacques[1], Cheryl. E. Peters[3], Aaron A. Goodarzi[1**], Linda E. Carlson[4**]

Author Affiliations:

[1]Robson DNA Science Centre, Departments of Biochemistry & Molecular Biology and Oncology, Charbonneau Cancer Institute, Cumming School of Medicine, University of Calgary, Calgary, Alberta, Canada; [2]Faculty of Medicine, University of Saskatchewan, Saskatoon, Saskatchewan, Canada; [3]Cancer Epidemiology & Prevention Research, Alberta Health Services and Departments of Oncology & Community Health Sciences, Cumming School of Medicine, University of Calgary, Alberta, Canada. [4]Division of Psychosocial Oncology, Department of Oncology, Charbonneau Cancer Institute, Cumming School of Medicine, University of Calgary, Calgary, Alberta, Canada.

*These authors contributed equally.
**Co-Corresponding authors are: A. Goodarzi (a.goodarzi@ucalgary.ca) and L. Carlson (l.carlson@ucalgary.ca)

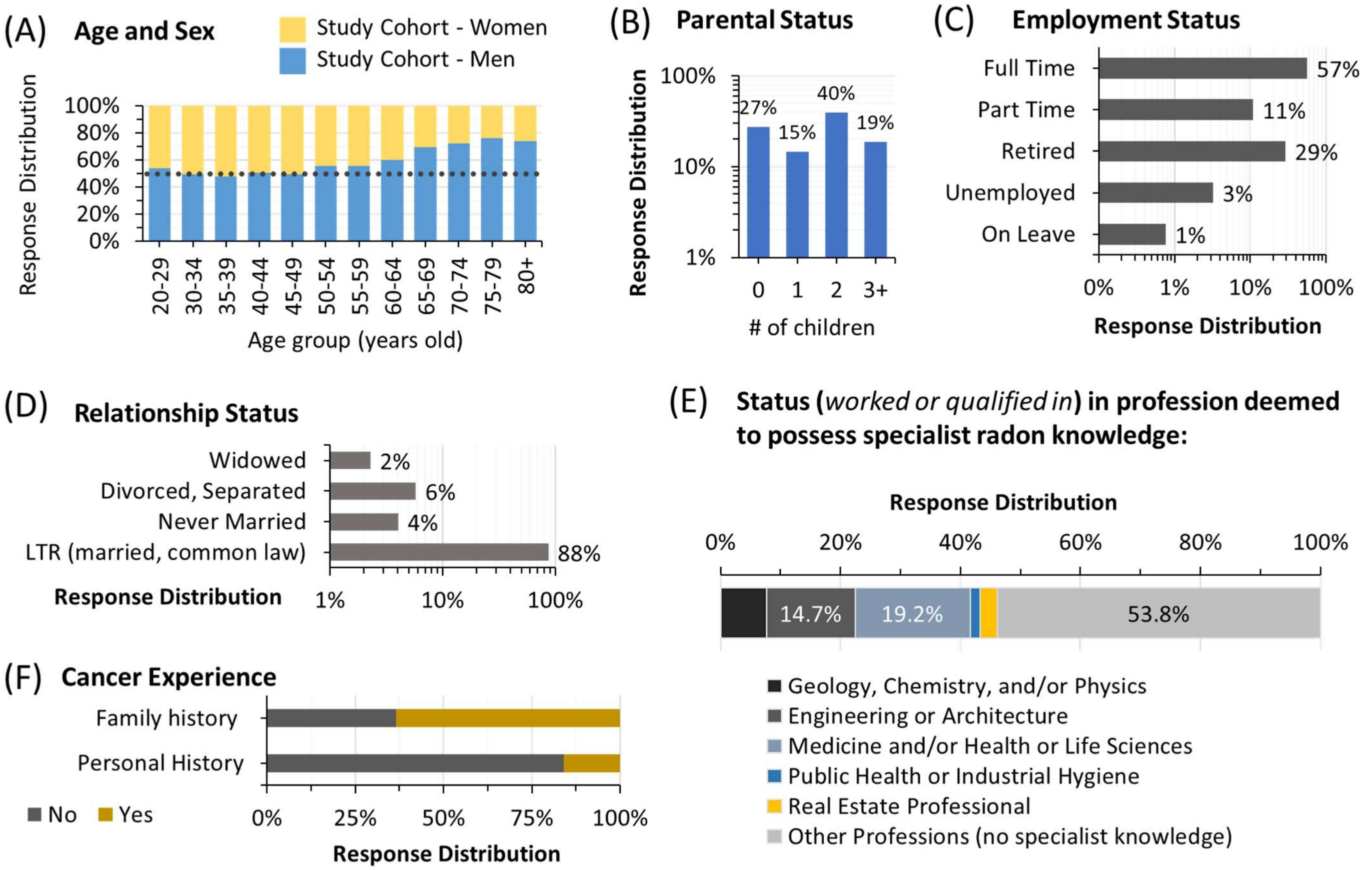

Supplemental Figure 1. Demographic distribution of study cohort. Panel A. Distribution of participants by age and sex. Panel B: Distribution of participants by parental status (number of children). Panel C: Distribution of participants by reported employment status. Panel D. Distribution of participants by relationship status. LTR = Long Term Relationship. Panel E: Distribution of participants by status (worked or qualified) in professions with or without the potential for specialist knowledge of radon, as listed. Panel F. Distribution of participants by experience with a cancer (of any type) diagnosis, where 'personal history' refers to the participant or an immediate member of participant's household has/had cancer, and 'family history' refers to a known incidence of cancer within the participants living or non-living family, excluding themselves and immediate members of household.

SECTION I. Survey Questionnaire

1. How did you FIRST hear about radon and its health hazards? (SELECT ONE)
    - Traditional News Media (TV, Print, Radio)
    - Social Media or Internet Search (Facebook, Twitter, Google, etc.)
    - Word of Mouth (your partner, friends, family, etc.)
    - Directly from a public health professional (doctor, scientist, etc.)
    - Directly from a private company
    - Other

2. After first hearing about radon and its health risks, where did you **NEXT** seek or obtain more information? (SELECT ALL THAT APPLY)
    - I did not seek more information.
    - Traditional News Media (TV, Print, Radio)
    - Social Media or Internet Search (Facebook, Twitter, Google, etc.)
    - Word of Mouth (your partner, friends, family, etc.)
    - Directly from a public health professional (doctor, scientist, etc.)
    - Directly from a private company
    - Other (if they select this a text box opens for them to type in details)

3. How knowledgeable would you say you are about how to decrease high radon levels found in home?
    - Extremely
    - Very
    - Moderately
    - Slightly
    - Not at all

4. How knowledgeable would you say you are about how radon gas can be found in homes?
    - Extremely
    - Very
    - Moderately
    - Slightly
    - Not at all

5. How knowledgeable would you say you are about how of radon gas in cancer risk?
    - Extremely
    - Very
    - Moderately
    - Slightly
    - Not at all

6. Once you felt you understood the role of radon gas in cancer risk and how it can be found in people’s homes, how did you feel?
    a. No strong feeling
    b. Relieved

- Extremely
- Very
- Moderately
- Slightly
- Not at all

c. Confidant (5 point Likert Scale as above)
d. Anxious (5 point Likert Scale as above)
e. Fearful (5 point Likert Scale as above)
f. Angry (5 point Likert Scale as above)
g. Disgusted (5 point Likert Scale as above)

7. Approximately how many times did you hear or read about radon, before deciding to get a radon test for your property(s)? [NUMERICAL RESPONSE]

8. Approximately how long was it from <u>first hearing about</u> radon to registering for or obtaining a radon test kit? (days, months, years)

9. How important were each of these factors in your decision to test for radon? (select all that apply)
   a. I was/am curious about the radon levels of my home
      - Very important
      - Important
      - Fairly Important
      - Slightly important
      - Not important
   b. I was/am concerned about the welfare of the residents of my home
      - (5 point Likert Scale as above)
   c. My partner, friends or family prompted me to test my home for radon
      - (5 point Likert Scale as above)
   d. My landlord prompted me / I prompted my landlord to test my home for radon
      - (5 point Likert Scale as above)
   e. Other

10. Did any of your friends, colleagues or relations decide to test their homes for radon, during the same period of time that you were testing your home?
    a. No (skip to next question)
    b. Yes
       I. How strongly was your decision to obtain a radon test for your home motivated by knowing that others in your social circle were testing for radon?
          - Extremely
          - Very
          - Moderately
          - Slightly
          - Not at all
       II. Were any of your friends, colleagues or relations (who tested for radon) motivated by knowing that YOU had radon tested / were testing YOUR house?
          - Yes, I am aware of some

- None that I know of

11. How long did it take you from the time you received your radon test, to setting it up in your home?
    - Less than a week
    - Less than a month
    - Within 3 months
    - Within 6 months
    - Within a year
    - Between 1-2 years
    - Between 2-4 years
    - More than 4 years
    - Did not deploy yet
    - Do not plan to deploy it/changed my mind

9. How much do you agree with the following statement; "*I am worried about radon's ability to cause serious illness in me in the future*" [7]
    - Completely disagree
    - Partly disagree
    - No opinion
    - Partly agree
    - Completely agree

10. How much do you agree with the following statement; "*I will remain healthy even if I'm exposed to radon due to my good general health status and physical resilience*" [7]
    - Completely disagree
    - Partly disagree
    - No opinion
    - Partly agree
    - Completely agree

11. What age were you at the time you decided to radon test your home (with our study)?

12. What was your biological sex at birth?
    - Female
    - Male
    - Other______
    - Prefer not to say

13. What was your employment status at the time you decided to obtain a radon test? [11]
    - Unemployed
    - Full Time
    - Part Time
    - Retired
    - Disability/Leave
    - Prefer not to say

14. Have you ever successfully obtained qualifications in and/or been employed in any of the following professions? (SELECT ALL THAT APPLY)
    - Radon Testing / Mitigation
    - Medicine and/or Health or Life Sciences
    - Earth (Geological) Sciences, Chemistry, and/or Physics
    - Public Health or Industrial Hygiene
    - Engineering or Architecture
    - Real Estate

15. What was your relationship status at the time you decided to obtain a radon test?
    - Single, never married
    - Single, divorced
    - Long term relationship, unmarried
    - Long term relationship, married or civil partnership
    - I was a widow or widower

16. How many children did you have at the time you decided to obtain a radon test? ([12])
    - 0
    - "0" (but I was/we were pregnant with a first child, or expecting to adopt)
    - 1
    - 2
    - 3
    - 4 or more

17. Were you aware of any family history of cancer at the time of obtaining a radon test? (Yes/No)

18. Had you ever been diagnosed with cancer at the time of obtaining a radon test? (Yes/No)

19. Not including yourself, had anyone else in the home been diagnosed with cancer at the time of obtaining a radon test? (Yes/No)

SECTION II. Examples of Radon Awareness Information

The 2020 Government of Canada Public Health Information Card on Radon.

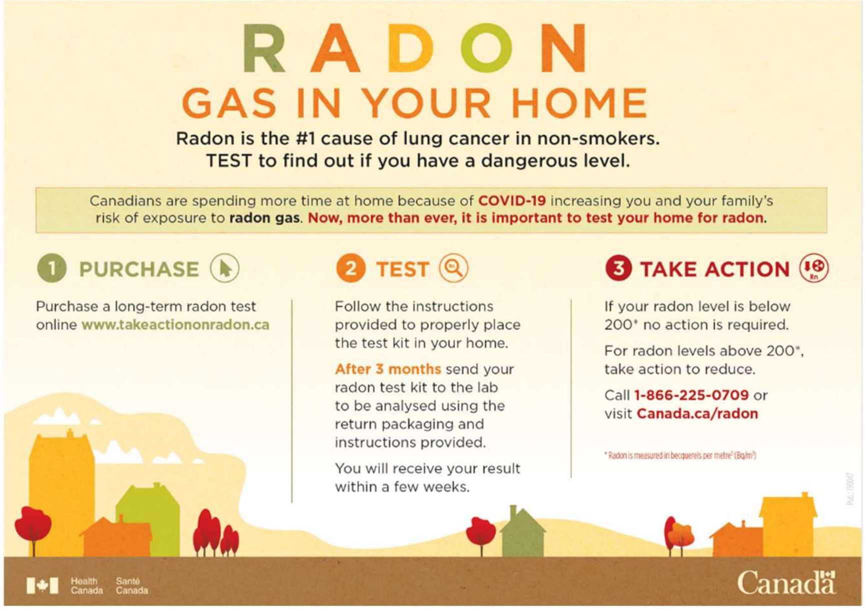

FROM: https://www.canada.ca/en/health-canada/services/environmental-workplace-health/reports-publications/radiation/radon-your-home-health-canada-2009.html

The 2020 USA Environmental Protection Agency webpage on Public Health Information of Radon.

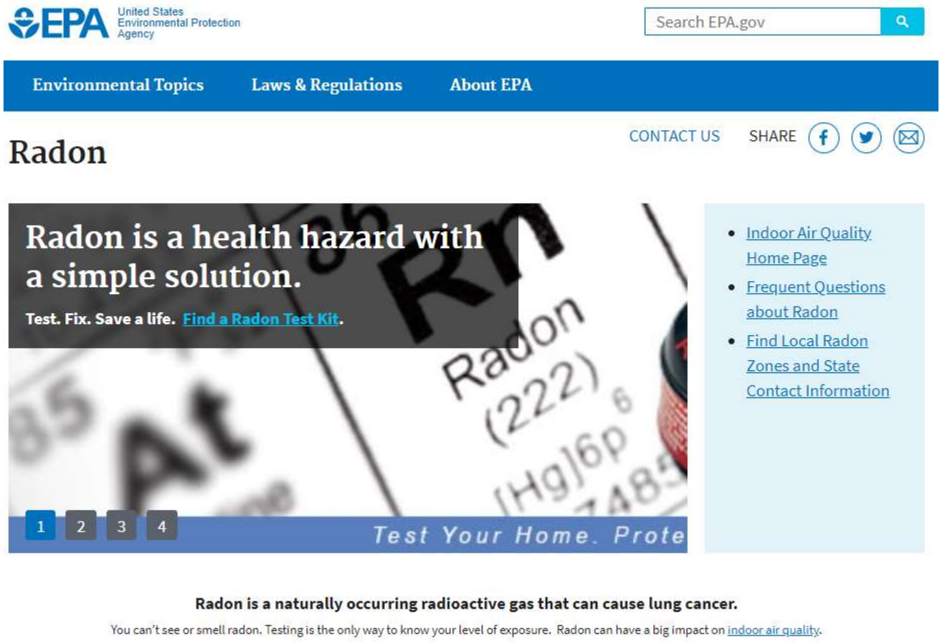

FROM: https://www.epa.gov/radon

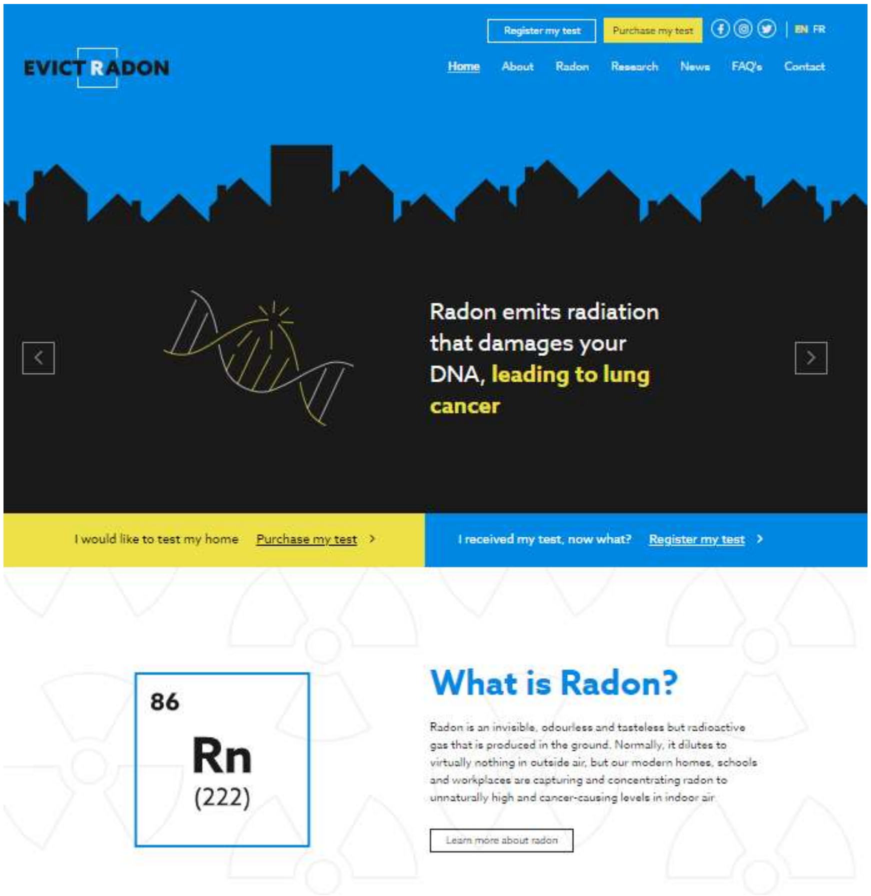

The 2020-2021 Canadian Evict Radon National Study Website Home Page From www.evictradon.org and associated social media accounts (Facebook, Twitter).

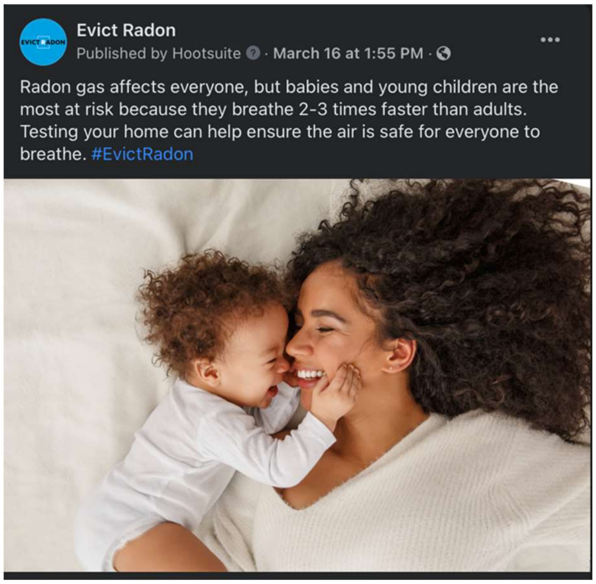

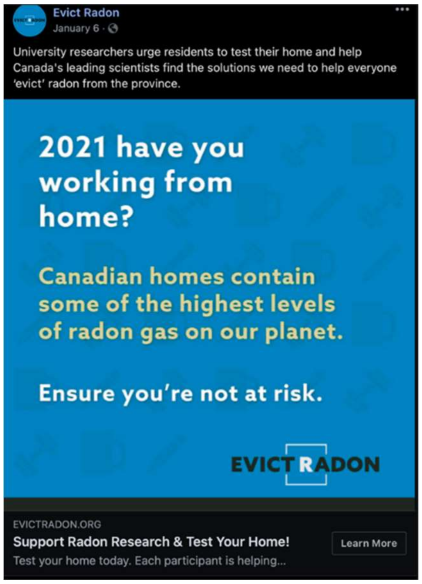

The 2020 Public Health England webpage of Public Health Information on Radon.

Public Health England

UKradon

Contact us Sign in to your account

Home | About radon | Sector guidance | Our services | About us | FAQ | Resources

Welcome to UKradon

Public Health England

Three steps to manage radon in buildings - check, measure, act

Every building has radon and in most areas the levels are low. Some buildings in "radon Affected Areas" have higher levels. Buildings in these areas should be tested for radon. High levels can be reduced by simple building works. There are three simple steps you can follow:

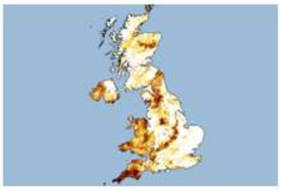

1. Check

Is your property in a radon Affected Area?

Find out more

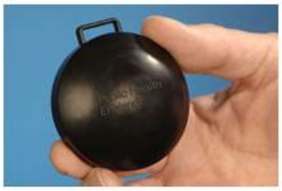

2. Measure

If you are in a radon Affected Area you should order a radon measurement pack for your home or workplace

Find out more

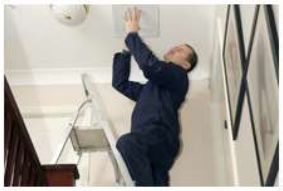

3. Act

If the radon level is high you should reduce it using simple building works

Find out more

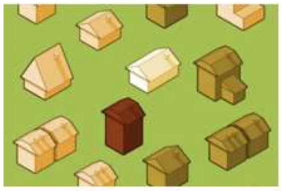

Radon information

You may also wish to view other topics about radon. This section covers what radon is, the health risks, maps of radon affected areas and many other topics.

More information

Radon measurement pack

Find out the yearly average radon level for a property and if it is above or below the Action Level.

Order a domestic pack

Order a workplace pack

A beginners guide to radon

This short animation covers: What is radon? Radon in homes, Radon risks, Radon areas, Radon reduction.

Radon at a glance

PHE Radiation Protection Services

PHE delivers radiological assessment consultancy services, training, expert information and advice and products.

Overview of all our services

FROM: https://www.ukradon.org/

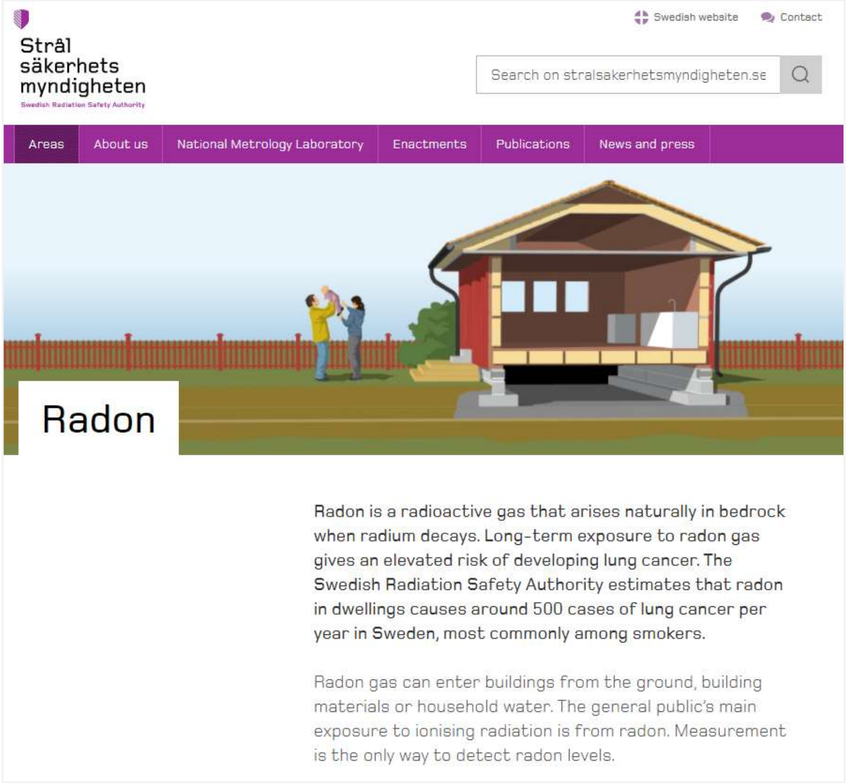

Strål säkerhets myndigheten
Swedish Radiation Safety Authority

Swedish website | Contact

Search on stralsakerhetsmyndigheten.se

Areas | About us | National Metrology Laboratory | Enactments | Publications | News and press

Radon

Radon is a radioactive gas that arises naturally in bedrock when radium decays. Long-term exposure to radon gas gives an elevated risk of developing lung cancer. The Swedish Radiation Safety Authority estimates that radon in dwellings causes around 500 cases of lung cancer per year in Sweden, most commonly among smokers.

Radon gas can enter buildings from the ground, building materials or household water. The general public's main exposure to ionising radiation is from radon. Measurement is the only way to detect radon levels.

The 2020 Swedish Radiation Safety Authority webpage of Public Health Information on Radon.

FROM: https://www.stralsakerhetsmyndigheten.se/en/areas/radon/

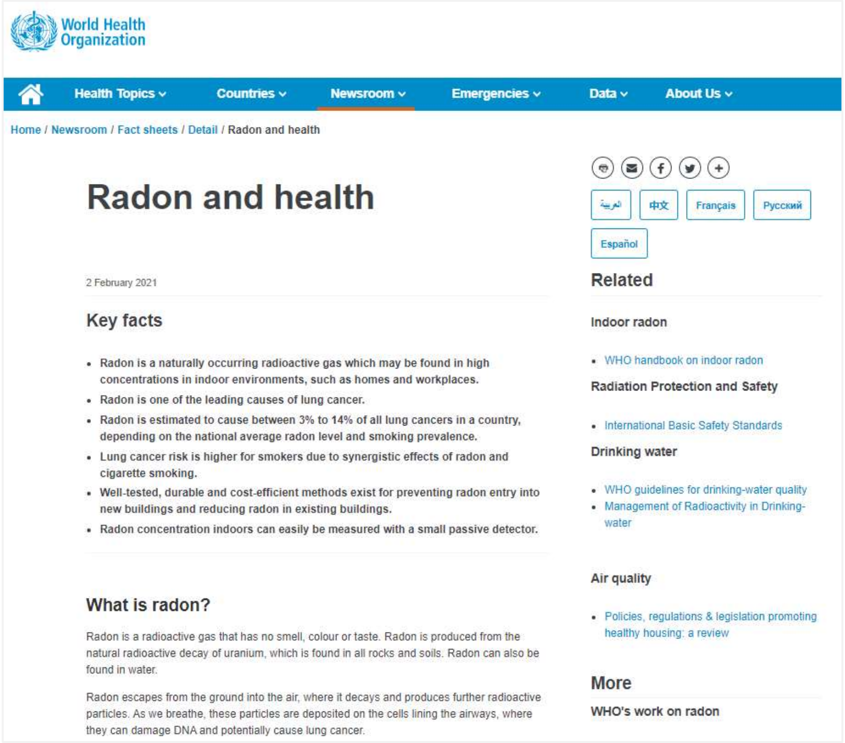

World Health Organization

Health Topics | Countries | Newsroom | Emergencies | Data | About Us

Home / Newsroom / Fact sheets / Detail / Radon and health

Radon and health

2 February 2021

Key facts

- Radon is a naturally occurring radioactive gas which may be found in high concentrations in indoor environments, such as homes and workplaces.
- Radon is one of the leading causes of lung cancer.
- Radon is estimated to cause between 3% to 14% of all lung cancers in a country, depending on the national average radon level and smoking prevalence.
- Lung cancer risk is higher for smokers due to synergistic effects of radon and cigarette smoking.
- Well-tested, durable and cost-efficient methods exist for preventing radon entry into new buildings and reducing radon in existing buildings.
- Radon concentration indoors can easily be measured with a small passive detector.

What is radon?

Radon is a radioactive gas that has no smell, colour or taste. Radon is produced from the natural radioactive decay of uranium, which is found in all rocks and soils. Radon can also be found in water.

Radon escapes from the ground into the air, where it decays and produces further radioactive particles. As we breathe, these particles are deposited on the cells lining the airways, where they can damage DNA and potentially cause lung cancer.

العربية | 中文 | Français | Русский | Español

Related

Indoor radon
- WHO handbook on indoor radon

Radiation Protection and Safety
- International Basic Safety Standards

Drinking water
- WHO guidelines for drinking-water quality
- Management of Radioactivity in Drinking-water

Air quality
- Policies, regulations & legislation promoting healthy housing: a review

More

WHO's work on radon

The 2020 World Health Organization webpage of Public Health Information on Radon.

FROM: https://www.who.int/news-room/fact-sheets/detail/radon-and-health

BREATHE
the lung association

Lung Health | Lung Diseases | Lung Research | Healthcare Providers | Support & Education | Get Involved

Home › Lung Health › Radon

Print Page A A A

Due to the increase in COVID-19 cases, our office is closed until further notice. Please see our news post for more information.

Radon

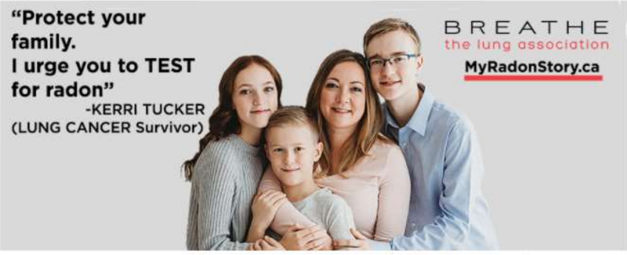

#MyRadonStory

What is radon?
Radon is a gas formed by the breakdown of uranium, a natural radioactive material found in all soil and rock.

Buy a Test Kit?

ORDER ONLINE

Shipping available anywhere in Canada.

Have a Test Kit?

START TEST

You must register your test at homeradontest.ca to receive your results.

The 2020 Canadian Lung Association (for province of Saskatchewan) webpage of Public Health Information on Radon.

FROM: https://www.lungsask.ca/lung-health/radon

BfS Bundesamt für Strahlenschutz

Deutsch | Sitemap | FAQ | Glossary | Contact

TOPICS | NEWS | MEDIA CENTRE | THE BFS

IONISING RADIATION

Environmental Radioactivity - Medicine - Occupational Radiation Protection - Nuclear Hazards Defence

Homepage › Topics › Ionising radiation › Radioactivity in the environment › Radon

- What is ionising radiation?
- Radioactivity in the environment
- Where does radioactivity occur in the environment?
- What is the level of natural radiation exposure in Germany?
- Air, soil and water
- Radon
- › What is radon?

Radon

Radon is a very mobile, radioactive noble gas that cannot be seen, smelled or tasted. It is a result of the radioactive decay of uranium. Uranium, for example, exists in the ground or in building materials.

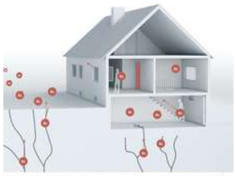

From the ground, radon enters the atmosphere and buildings. Outdoors, it immediately mixes with the ambient air, so that the radon concentration there is low. Indoors, however, high radon concentrations can be reached.

Breathing an enhanced amount of radon and its radioactive decay products over a longer period of time increases the risk of getting lung cancer. It is often sufficient to take small measures to clearly reduce the radon content and thus the risk of disease.

The Radiation Protection Act commits the state, employer, and developers to take measures for the protection against radon.

The 2020 German Federal Office for Radiation Protection webpage of Public Health Information on Radon.

FROM: https://www.bfs.de/EN/topics/ion/environment/radon/radon_node.html
